# Volar Dislocation of the Fourth and Fifth Carpometacarpal Joint Associated with Hamate Hook Fracture: A Case Report and Literature Review

**DOI:** 10.1155/2020/6301692

**Published:** 2020-03-03

**Authors:** Natsumi Saka, Hirotada Matsui, Hideki Tsuji

**Affiliations:** ^1^Orthopaedic Trauma Center, Sapporo Tokushukai Hospital, Sapporo, Hokkaido, Japan; ^2^Department of Orthopaedics, Teikyo University School of Medicine, Tokyo, Japan

## Abstract

We report a case of volar fourth and fifth carpometacarpal (CMC) joint dislocation complicated by a hamate hook fracture. The CMC joint was reduced in a closed fashion and temporally fixed with Kirschner wires. Using intraoperative computed tomography, the displaced fracture of the hamate hook was reduced by open reduction and internal fixation and fixed with a screw. We suggest that this rare injury was caused by the over contraction of the flexor carpi ulnaris and avulsion force from the ligamentous structure around the pisiform, hamate, and metacarpal bones.

## 1. Introduction

Dislocation of the carpometacarpal (CMC) joint is a relatively rare injury, and volar dislocation of the CMC joint is less common than dorsal CMC joint dislocation. Nalebuff reported two types of volar dislocations of the fifth CMC joint: volar radial or volar ulnar, depending on the type of ruptured ligament [1]. Fracture of the hamate hook is also an uncommon injury, usually seen in athletes and caused by direct compression [2]. The purpose of this study was to present a case of volar dislocation of the fourth and fifth CMC joint with a hamate hook fracture and to discuss the mechanism of this injury. Careful assessment of radiographic findings is of paramount importance when considering the treatment strategy for this injury.

## 2. Case Report

**Figure 1:**
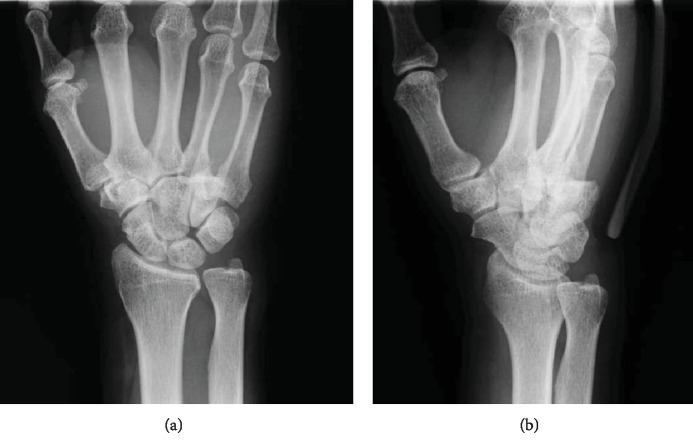
Initial radiograph showing the proximal displacement of the fourth and fifth metacarpal bones. (a) Posteroanterior view. (b) Oblique semisupinated view.

**Figure 2:**
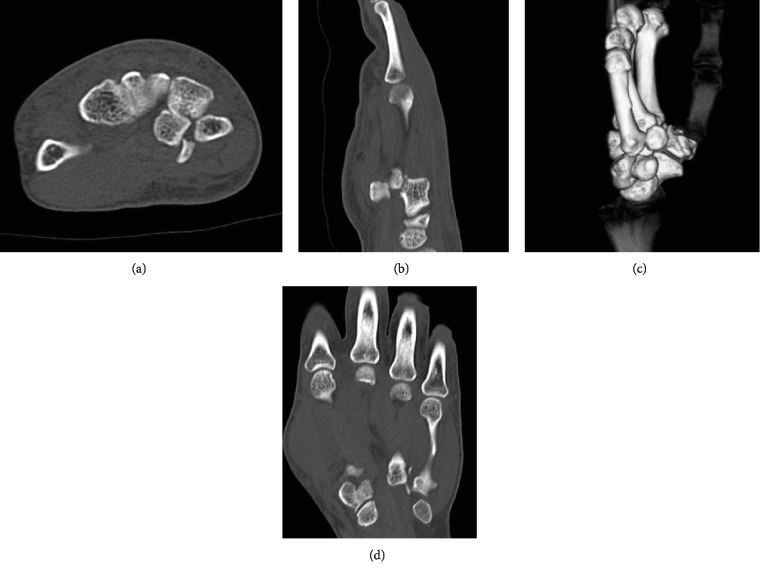
CT scan showing the carpometacarpal bases incarcerated at the fracture gap of the hamate hook at the fracture site. (a) Axial view. (b) Sagittal view. (c) Three-dimensional view of the hand. (d) Coronal view of the CT scan also shows the avulsion fracture between the base of the fourth and fifth metacarpal bones.

A 60-year-old man fell from a staircase and landed on the floor with his right wrist hyperextended. He had no history of hand or wrist injury. He presented to the nearby clinic complaining of severe pain and swelling of the right hand. A diagnosis of fourth and fifth CMC joint dislocation was made by radiography (Figures 1(a) and 1(b)). Closed reduction was not successful, and he was referred to our clinic on the day after the injury. Physical examination revealed significant swelling of the hand. Sensation over the median and ulnar nerve area was intact, as was abductor pollicis brevis and interossei function. Computed tomography (CT) revealed a fracture of the hamate hook associated with volar ulnar dislocation of the fourth and fifth CMC joint, and the base of the dislocated metacarpal was incarcerated between the hook and body of the hamate (Figures 2(a)–2(c)). An avulsion fracture was noted between the base of the fourth and fifth metacarpal bones (Figure 2(d)).

**Figure 3:**
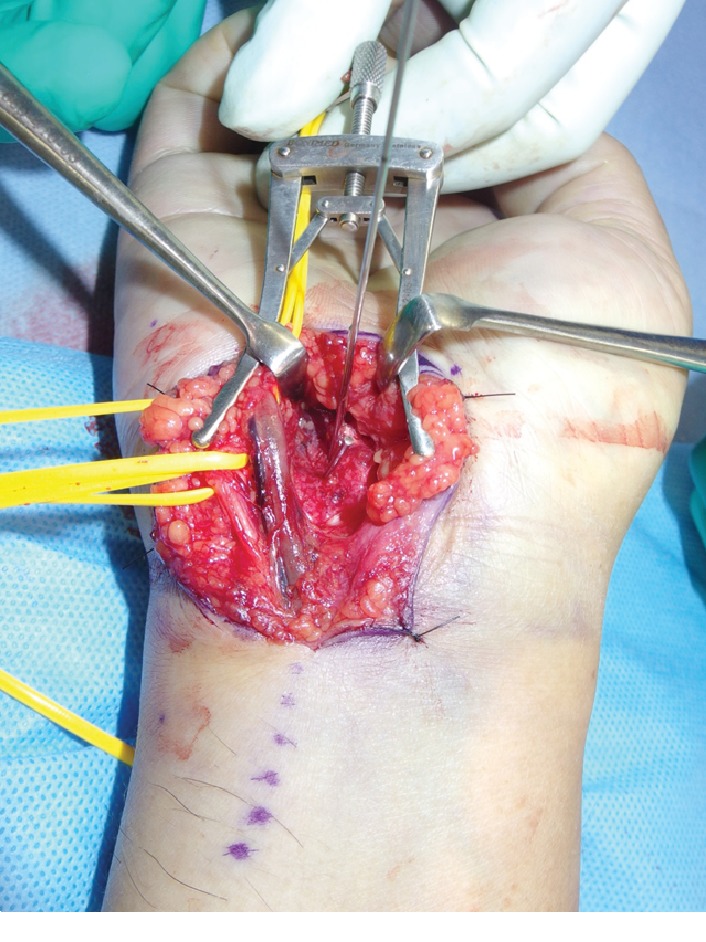
Intraoperative findings.

**Figure 4:**
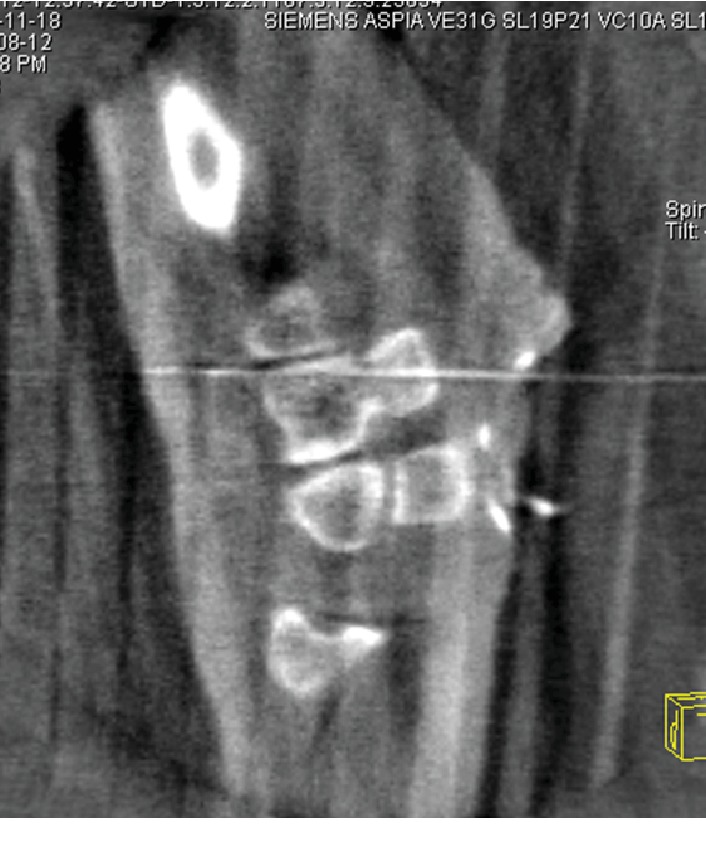
Intraoperative CT scan (sagittal view) showing the anatomical reduction of the hamate hook.

**Figure 5:**
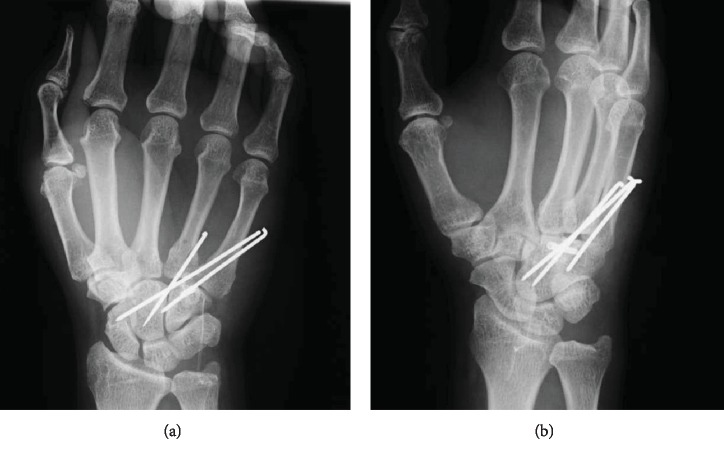
Postoperative radiograph. (a) Posteroanterior view. (b) Oblique semisupinated view.

**Figure 6:**
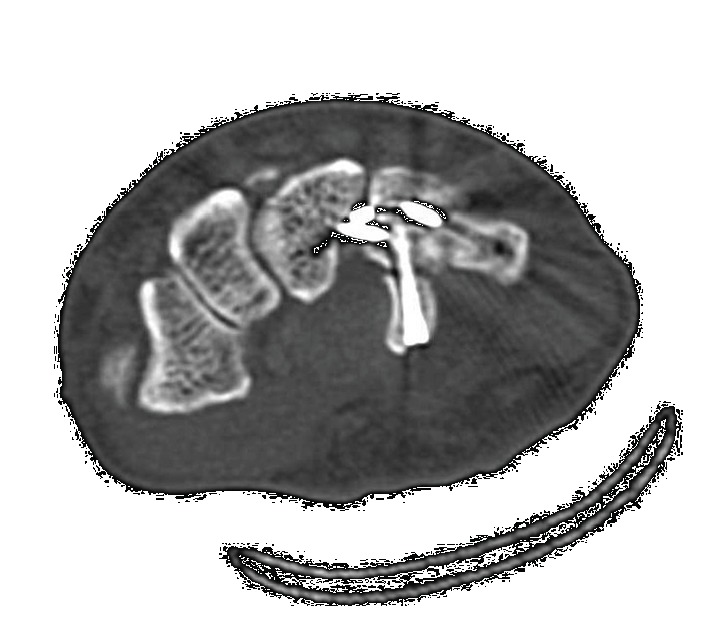
Postoperative CT scan showing the reduction and appropriate placement of the screw.

**Figure 7:**
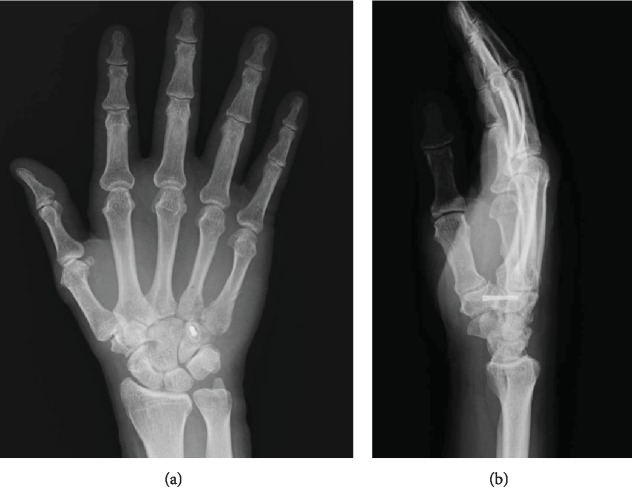
Radiographs after the removal of the K-wires showing the maintenance of the reduction of CMC joint. (a) Posteroanterior view. (b) Oblique semisupinated view.

Dislocation of the CMC joint was performed by longitudinal traction under sedation. The patient underwent open reduction and internal fixation of the hamate hook and percutaneous fixation of the CMC joint on the following day. The surgery was performed under general anesthesia. Surgical exposure was achieved with a longitudinal skin incision made between the hamate hook and pisiform, which was prolonged proximally by a palmer crease in a zigzag fashion. Guyon's canal was released for the exposure and protection of the ulnar artery and nerve. The pisiform and hamate hook was identified. The fracture site of the hamate hook was located using a longitudinal incision of the palmar carpal ligament (Figure 3). It was difficult to acquire good exposure of the fracture because of its depth in the surgical field. Therefore, to examine the reduction of the fracture, we used intraoperative CT after provisional fixation of the fracture site (Figure 4). After confirmation of the reduction, fixation was performed with a headless compression screw (Acutrak2 micro®, Acumed). The fourth and fifth CMC joint were temporally fixed percutaneously from the metacarpal bones to the carpal bones, respectively, using 1.5 mm Kirschner wires (K-wires; Figures 5(a) and 5(b)). Reduction and appropriate placement of the screw were confirmed postoperatively using CT (Figure 6). Immediate exercise of the fingers and wrist motion was allowed under a protective splint, and the K-wires were removed 7 weeks after surgery. A radiograph demonstrated the maintenance of the reduction after the removal of the K-wires (Figures 7(a) and 7(b)).

**Figure 8:**
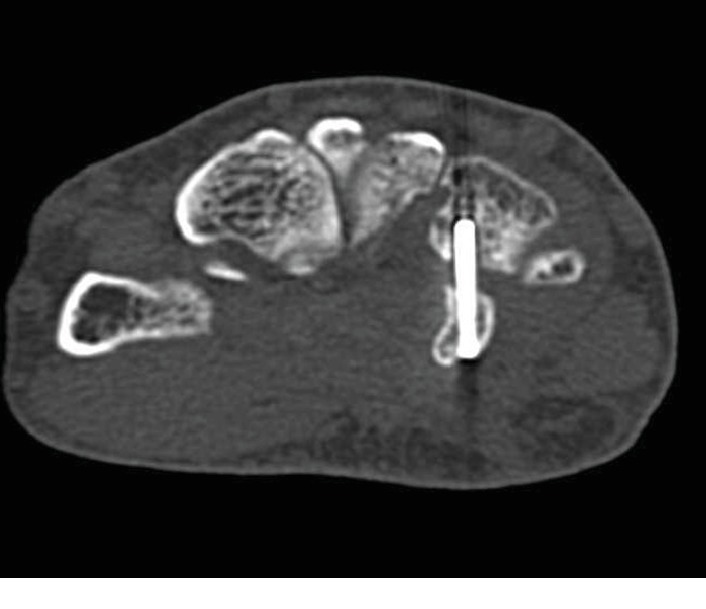
CT scan performed 3 months postoperatively showing the gap at the fracture site.

**Figure 9:**
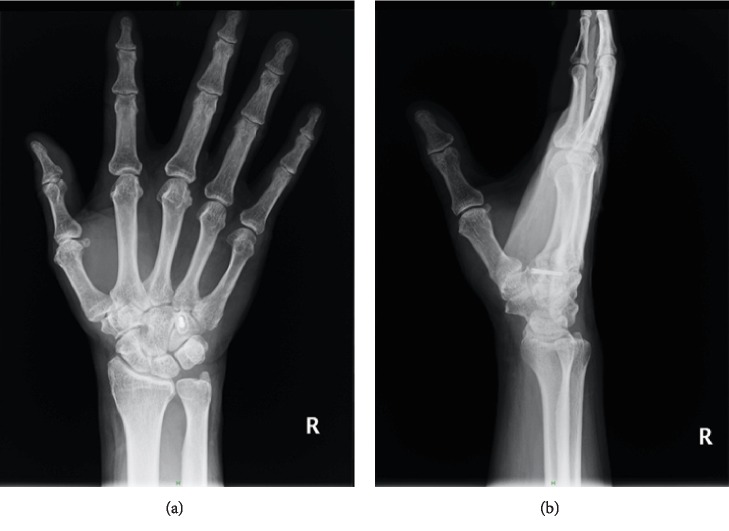
Radiograph 2 years after the surgery. (a) Posteroanterior view. (b) Oblique semisupinated view.

Three months after the operation, CT revealed a gap of the fracture site at the hamate hook (Figure 8). However, the patient did not have tenderness at the fracture site, and no secondary surgery was performed. Two years after the injury, the patient's active range of motion of the wrist was an extension of 75° and flexion of 60°. The Disability of the Arm, Shoulder, and Hand score was 0. He had no signs of rupture or irritation of the flexor digitorum profundus (Figure 9). The patient was informed that data from his case would be submitted for publication, and he provided consent.

## 3. Discussion

Several studies have reported volar joint dislocation of the fifth CMC joint, mostly without accompanying fracture of the hamate hook [1, 3, 4]. Nalebuff reported two types of volar CMC joint dislocations of the fifth finger: volar radial or volar ulnar, according to the pattern of ligamentous injury [1]. The pisometacarpal, carpometacarpal (volar fifth metacarpal hook of the hamate ligament), and metacarpal interosseous ligaments (volar fourth metacarpal ulnar base–fifth metacarpal radial base ligament) are attached to the base of the fifth finger. Volar radial dislocation is accompanied by rupture of all three ligaments, whereas volar ulnar dislocation is accompanied by rupture of the CMC and metacarpal interosseous ligaments, leaving the pisometacarpal ligament intact.

**Figure 10:**
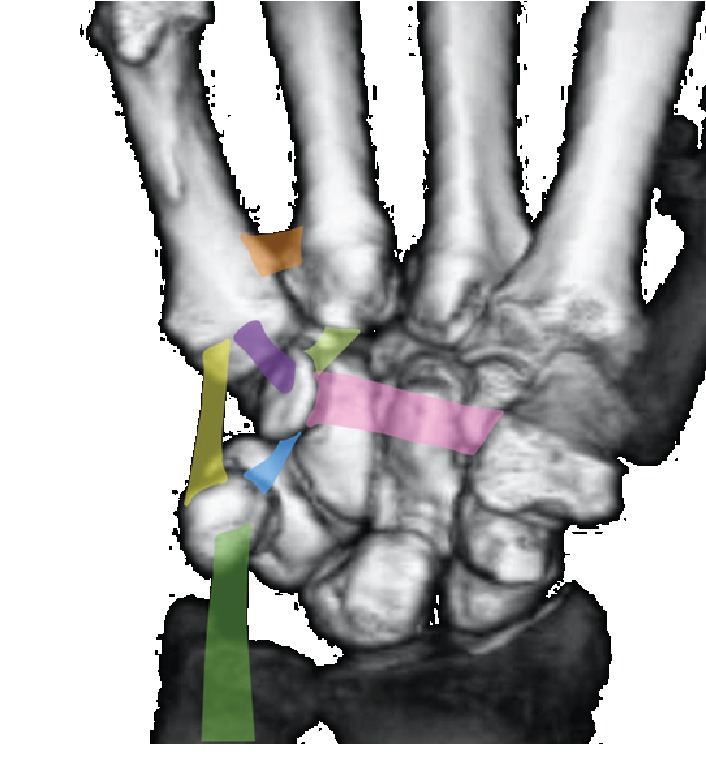
Ligamentous structure around the metacarpal bones, hamate hook, and pisiform. 1: flexor carpi ulnaris. 2: volar fifth metacarpal–pisiform ligament (pisometacarpal ligament). 3: volar fifth metacarpal–hamate hook ligament. 4: volar fourth metacarpal ulnar base–hamate hook ligament. 5: metacarpal interosseous ligament (volar fourth metacarpal ulnar base–fifth metacarpal radial base ligament). 6: pisohamate ligament. 7; transverse carpal ligament.

In our case, the exact mechanism of the hamate hook fracture associated with the volar dislocation of the fourth and fifth CMC joint was unclear; however, we suggest that this injury was an avulsion injury, based on previously published studies. Garcia-Elias et al. reported a hamate hook fracture with volar dislocation of the fifth CMC joint and pointed out that this injury was caused as an avulsion injury from the ligamentous structure around the pisiform [5]. These authors suggested that the force from the flexor carpi ulnaris is transmitted distally to the pisohamate and pisometacarpal ligaments through the pisiform. They also indicated that tension on the pisohamate ligament causes an avulsion fracture of the hamate hook, and tension on the pisometacarpal ligament produces volar dislocation of the fifth CMC joint (Figure 10). Jackson et al. also reported a hamate hook fracture in an individual participating in clay shooting and suggested that the injury was caused by the avulsion force from the pisohamate ligament [6]. Our case is similar to the injury described by Garcia-Elias et al. but was accompanied by an additional fourth CMC joint dislocation and avulsion fracture between the base of the fourth and fifth metacarpal. Therefore, we speculated that hamate hook fracture and volar fifth CMC dislocation were caused by the avulsion force to the pisohamate and pisometacarpal ligament, as suggested by Garcia-Elias et al. With regard to the fourth CMC joint dislocation, we speculated that it was caused by the traction from the volar fourth metacarpal ulnar base–hamate hook ligament (Figure 10).

To provide an anatomical and biomechanical background for this hypothesis, Pevny et al. showed that the pisohamate and pisometacarpal ligaments are much thicker and stronger than other soft-tissue attachments around the pisiform [7]. Rayan et al. proved that the pisometacarpal and pisohamate ligaments are the primary stabilizers of the pisiform [8]. Based on these anatomical studies, it can be speculated that avulsion force to the pisiform is transmitted to its primary stabilizer. Avulsion force by the pisometacarpal ligament leads to ulnar volar displacement of metacarpal, and avulsion force by the pisohamate ligament leads to the fracture of the hook of the hamate. Yoshida et al. conducted a biomechanical study using a cadaver to simulate a blow to the ulnar side of the hand with the wrist extended [9]. They showed that a fifth metacarpal volar base fracture and hamate hook fracture are attached to the volar fifth metacarpal–hamate hook ligament and concluded that the pathomechanical etiology of this injury is avulsion. This supports the hypothesis of our case, namely, that the metacarpal–hamate hook ligament caused the avulsion injury.

In terms of treatment of hamate hook fractures, most injuries of this type, which are typically caused by repetitive microtrauma or blunt trauma to the palm of the hand from playing golf or baseball, are treated by hook excision. In the report by Gunther et al., the fracture was left untreated, resulting in nonunion without symptoms [10]. Another report from Garcia-Elias et al. recommended open reduction and internal fixation of the hamate hook, which resulted in stabilization of the pisohamate ligament and pisiform [5]. The hook was successfully fixed with K-wires, and union was demonstrated on radiography.

In our case, open reduction and internal fixation of the fractured hook was performed, taking into account the mechanism of this injury. In contrast to the usual type of hamate hook fracture, we noted a gap between the hook and body of the hamate. An intraoperative three-dimensional CT scan was helpful for obtaining anatomical reduction of the fracture site. The fracture resulted in nonunion, which may have been related to the initial displacement and residual instability around the hamate hook, since the HCS screw length did not exceed the more than half of the hamate body.

This is the first report to describe volar dislocation of the fourth and fifth CMC dislocation with a hamate hook fracture and its relationship to the ligamentous structure around the hamate hook. Our case indicates the importance of traction force in the strategic treatment of this injury.
